# Retraction: miR-384 induces apoptosis and autophagy of non-small cell lung cancer (NSCLC) cells through the negative regulation of Collagen alpha-1(X) chain (COL10A1) gene

**DOI:** 10.1042/BSR20181523_RET

**Published:** 2026-03-26

**Authors:** Min Zheng, Qingkui Guo, Ye Xu, Ning Wang, Wen Zhao

**Affiliations:** Department of Thoracic Surgery, Tongren Hospital, Shanghai Jiao Tong University, School of Medicine, Shanghai 200336, P.R. China

**Keywords:** Cell apoptosis, Cell autophagy, COL10A1, miR-384, Non-small cell lung cancer

This article is being retracted from *Bioscience Reports* at the request of the Editor-in-Chief and the Editorial Board. This follows the receipt of a notification from a reader, alerting the Editorial Board to concerns over the potential use of inappropriate primers.

Upon investigation, the Editorial Office identified what appears to be two regions of duplication. Specifically, one of the cells in the Figure 14A miR-384 mimic panel has high similarity to a cell in the miR-384 inhibitor + siRNA-COL10A1 panel. In addition, the Figure 13A NC panel from this article appears to be the same as the Figure 4A Blank panel in the article Tang et al. (2019) https://doi.org/10.1002/jcb.28600.

The authors have been contacted with regard to the retraction. They stated that the U6 snRNA was not amplified using inappropriate primers and disagreed with the similarities in Figure 14A. They did not respond to further contact regarding the Figure 13A panel similarity.

Given the extent of the issues raised, the Editorial Board stands by the decision to retract the article. The authors disagree to the retraction.

